# Prediction of outcome in patients with ARDS: A prospective cohort study comparing ARDS-definitions and other ARDS-associated parameters, ratios and scores at intubation and over time

**DOI:** 10.1371/journal.pone.0232720

**Published:** 2020-05-06

**Authors:** Wolfgang Huber, Michael Findeisen, Tobias Lahmer, Alexander Herner, Sebastian Rasch, Ulrich Mayr, Petra Hoppmann, Juliane Jaitner, Rainer Okrojek, Franz Brettner, Roland Schmid, Paul Schmidle

**Affiliations:** 1 Medizinische Klinik und Poliklinik II, Klinikum rechts der Isar der Technischen Universität München, München, Germany; 2 Klinik für Pneumologie, Gastroenterologie, Internistische Intensiv- und Beatmungsmedizin, München Klinik Harlaching, München, Germany; 3 Medizinische Klinik und Poliklinik I, Klinikum rechts der Isar der Technischen Universität München, München, Germany; 4 Abteilung Intensivmedizin, Krankenhaus Barmherzige Brüder, München, Germany; Duke University, UNITED STATES

## Abstract

**Background:**

Early recognition of high-risk-patients with acute respiratory distress syndrome (ARDS) might improve their outcome by less protracted allocation to intensified therapy including extracorporeal membrane oxygenation (ECMO). Among numerous predictors and classifications, the American European Consensus Conferenece (AECC)- and Berlin-definitions as well as the oxygenation index (OI) and the Murray-/Lung Injury Score are the most common. Most studies compared the prediction of mortality by these parameters on the day of intubation and/or diagnosis of ARDS. However, only few studies investigated prediction over time, in particular for more than three days.

**Objective:**

Therefore, our study aimed at characterization of the best predictor and the best day(s) to predict 28-days-mortality within four days after intubation of patients with ARDS.

**Methods:**

In 100 consecutive patients with ARDS severity according to OI (mean airway pressure*F_i_O_2_/p_a_O_2_), modified Murray-score without radiological points (Murray_mod), AECC- and Berlin-definition, were daily documented for four days after intubation. In the subgroup of 49 patients with transpulmonary thermodilution (TPTD) monitoring (PiCCO), extravascular lung water index (EVLWI) was measured daily.

**Primary endpoint:**

Prediction of 28-days-mortality (Area under the receiver-operating-characteristic curve (ROC-AUC)); IBM SPSS 26.

**Results:**

In the totality of patients the best prediction of 28-days-mortality was found on day-1 and day-2 (mean ROC-AUCs for all predictors/scores: 0.632 and 0.620). OI was the best predictor among the ARDS-scores (AUC=0.689 on day-1; 4-day-mean AUC = 0.625). AECC and Murray_mod had 4-day-means AUCs below 0.6. Among the 49 patients with TPTD, EVLWI (4-day-mean AUC=0.696) and OI (4-day-mean AUC=0.695) were the best predictors. AUCs were 0.789 for OI on day-1, and 0.786 for EVLWI on day-2. In binary regression analysis of patients with TPTD, EVLWI (B=-0.105; Wald=7.294; p=0.007) and OI (B=0.124; Wald=7.435; p=0.006) were independently associated with 28-days-mortality. Combining of EVLWI and OI provided ROC-AUCs of 0.801 (day-1) and 0.824 (day-2). Among the totality of patients, the use of TPTD-monitoring „per se“ and a lower SOFA-score were independently associated with a lower 28-days-mortality.

**Conclusions:**

Prognosis of ARDS-patients can be estblished within two days after intubation. The best predictors were EVLWI and OI and their combination. TPTD-monitoring „per se“ was independently associated with reduced mortality.

## Introduction

A reduction in mortality of patients with acute respiratory distress syndrome (ARDS; [1]) has been shown for low-tidal volume ventilation [2], prone positioning [3–5] and in one study on neuro-muscular blocking agents (NMBA) [6]. Two RCTs suggest a potential to improve outcome by ECMO in selected patients [7, 8]. Nevertheless, mortality of ARDS is about 40% [1, 9, 10]. Protracted recognition or even complete non-recognition of ARDS at all contributes to its high mortality [1, 10, 11]. ARDS remains unrecognized in two of three patients at the time of fulfillment of the ARDS criteria [1]. These findings suggest a low acceptance and/or sensitivity of the current definition.

ARDS is a *syndromic disease* without a sensitive and specific diagnostic test [12]. About 50 years after the first definition of ARDS and several modifications like the American-European Consensus Conference (AECC; [13]) also the most recent “Berlin-definition” is a matter of debate [14, 15]. AECC- and Berlin-definition are predominantly based on p_a_O_2_/F_i_O_2_ and neglect the impact of pulmonary compliance and other markers on the outcome of ARDS [16].

In addition to consensus-definitions several “informal” scores emerged such as the Murray (Lung Injury Score (LIS); [17]) which is based on predefined categories of p_a_O_2_/F_i_O_2_, PEEP, lung compliance and chest X-ray.

The combination of mean airway-pressure (P_maw) with p_a_O_2_/F_i_O_2_ defines the oxygenation-index (OI = P_maw * F_i_O_2_ * 100 / p_a_O_2_). Several studies demonstrated better prognostic capabilities of OI compared to pO_2_/F_i_O_2_ [18–20].

Furthermore, extravascular lung water index EVLWI has been suggested as ARDS-defining criterion [20–26].

Sensitive, specific and early diagnosis of ARDS is important to improve timing and allocation to specific interventions such as PP, NMBAs and ECMO [27]. Regarding side effects and resources required, optimized indication of these interventions is of high clinical and socio-economic interest. There is consensus that strategies to improve the effectiveness of ECMO are crucial. These strategies include an optimized patient selection. To optimize timing, a too early intervention in patients not in need for ECMO should be avoided. On the other hand, a protracted initiation of ECMO in a rescue-setting results in poor outcome [28].

Only few studies included systematic, repeated and early comparison of the predictive capacities of ARDS-definitions and scores regarding mortality (Table 1).

**Table 1:** Summary of studies comparing repeated prediction of outcome in acute respiratory distress syndrome ARDS.

Reference	Setting; Number of patients	Predictors	EVLWI available	No. of measurements	Main result
	Endpoint				
				No. of days	
Villar et al. [29]	ARDS	p_a_O_2_/F_i_O_2_	no	1 measurement	Predictive regression model (including age, P_plat, and p_a_O_2_/F_i_O_2_) and APACHE- • II significantly predict ICU-mortality. ROC-AUCs: Derivation cohort: • model: 0.725 - 0.810 • - APACHE-II: 0.620 - 0.695
	Deriviation: 170 patients			on day of fulfillment of ARDS APACHAPACHE	
		Plateu-pressure (P_plat)			
				Day-1 only	
		Age			
	Validation: 50 patients ICU-mortality	APACHE-II			
Kao et al. [30]	Severe acute respiratory failure (acute respiratory failure with >24h of MV	SOFA (day-1)	no	1 day (SOFA)	OI in the first 3 days of mechanical ventilation and high SOFA independently predict mortality.
				3 days (PaO_2_/F_i_O_2_)	
					ROC-AUCs: SOFA-score (day 1): 0.647
		p_a_O_2_/F_i_O_2_, (day 1,3)		3 days (OI)	
		OI (day 1, 3)			OI (day 3): 0.724
		Change of OI within the first 3 days			
	100 patients				
	Hospital mortality				
Dechert et al. [31]	Multicenter study (ALVEOLI database)	Day 1-4: Age, OI, age adjusted OI (AOI)	no	4 measurements	Deriviation cohort: Age: AUC=0.67 (day 1), similar results days 2-4
	541 patients	Day 1: p_a_O_2_/FiO_2_, age + p_a_O_2_/FiO_2_			
				4 days	OI: AUC=0.61 (day 1), similar results on days 2-4
					AOI (day 1-4): AUC: 0.73, 0.70, 0.70, 0.74
	28-days-mortality				p_a_O_2_/F_i_O_2_: AUC=0.42 (day 1)
					Age + PaO_2_/F_i_O_2_: AUC=0.52 (day 1)
					Validation cohorts
					FACCT: AOI (day 1-4): AUC: 0.70, 0.72, 0.73, 0.72
					ARMA: AOI (day 1-4): AUC: 0.74, 0.78, 0.77, 0.76
Balzer et al. (32)	ICU	AECC, Berlin, p_a_O_2_/F_i_O_2_, OI	no	442 patients	OI better predicts mortality compared to p_a_O_2_/F_i_O_2_, AECC or Berlin.
	Hospital mortality				
				7 measurements	OI is an independent predictor in the final model of regression analysis.
				7 days	Best early prediction on days 3 and 4.
Own study	General ICU	AECC, Berlin, LIS, OI, EVLWI	yes (49/99)		Best prediction on day 2.
	99 patients				
	49/99 patients with PiCCO and EVLWI			4 measurements	EVLWI and OI are independently associated with mortality.
					Similar impact and cut-offs of EVLWI and OI in the multivariate analysis.
	28-days-mortality				
				4 days	Sum of EVLWI and OI on day 2: ROC-AUC of 0.824
					A cut-off of 19 for EVLWI (mL/kg) + OI (cmH_2_O/mmHg) on day 2 provided a sensitivity of 71% and a specificity of 79% to predict 28d-mortality.
					Sum EVLWI+OI+SOFA on day-2 provided a ROC-AUC of 0.856.

ARDS: Acute Respiratory Distress Syndrome; ICU: Intensive Care Unit; MV: Mechanical ventilation; EVLWI: Extra-vascular Lung Water Index; APACHE-II: Acute Physiology And Chronic Health Evaluation; SOFA: Sequential Organ Failure Assessment; OI: Oxygenation Index; LIS: Lung Injury Score; AECC: American European Consensus Conference; ROC-AUC: Receiver-operating characteristics area under the curve

Therefore, we compared the early prediction of 28-days-mortality by AECC- and Berlin-definitions of ARDS, by OI, a modified Murray-score and—if available—by EVLWI in 100 ICU-patients with ARDS.

## Materials and methods

### Study design

The study was conducted in a general ICU of a university hospital between May 2015 and September 2016. The protocol was approved by the institutional review board (Ethikkommission der Fakultät für Medizin der Technischen Universität München; 343/18 S) and registered (ISRCTN32938630). The need for informed consent was waived due to the observational design.

### Data availability statement

Due to ethical and legal restrictions imposed by Ethikkommission der Fakultät für Medizin der Technischen Universität München, confidential data are available upon request. To receive anonymized data, readers are welcome to contact the corresponding author (Prof. Dr. Wolfgang Huber, Medizinische Klinik und Poliklinik II, Klinikum rechts der Isar der Technischen Universität München, Ismaninger Strasse 22, D-81675 München, Germany. Fax: 0049-89-4140-4808. E-mail: wolfgang.huber@tum.de). Professor Dr. Georg Schmidt, an affiliate of Ethikkommission der Fakultät für Medizin der Technischen Universität München, may be contacted at gschmidt@tum.de).

100 consecutive patients with ARDS according to the Berlin-definition [33] were included. No patients fulfilling this criterion were excluded. OI as well as grading according to the AECC- (acute lung injury (ALI), ARDS) and Berlin-definitions (mild, moderate, severe) of ARDS, modified Murray-score without radiological points (Murray_mod) were daily documented for four days after intubation and correlated with 28d-mortality. We did not include the radiological sub-score in the Murray-score, since the use of radiological assessment for the Murray-score has been questioned [34].

Irrespectively of the study, 49 patients were equipped with transpulmonary thermodilution (TPTD) monitoring (PiCCO; Pulsion Medical Systems SE; Feldkirchen, Germany) on the day of intubation. In these patients, EVLWI was documented daily. TPTD using the PiCCO-2-device was performed as described previously [20].

### Statistics and endpoints

There were two major goals of these analyses:

1. To characterize the best early pulmonary predictor of 28-days-mortality in patients with ARDS.
2. To characterize the best day(s) for early prediction of 28-days-mortality.

Primary endpoint: ROC-AUCs (Receiver-operating-characteristic areas under the curve) regarding the prediction of 28-days-mortality by AECC-definition, Berlin-definition, OI, Murray_mod were calculated on the 1st, 2nd, 3rd and 4th day after intubation.

In the subgroup with TPTD-monitoring, also EVLWI was investigated as potential predictor of 28-days-mortality (ROC-AUCs).

Secondary endpoints: Since outcome of patients with ARDS is strongly associated with non-pulmonary organ impairment [35], we also investigated the prediction of 28-days-mortality by APACHE-II and SOFA.

To account for interactions and potential independent associations of several predictors with outcome, we performed three binary regression analyses (Wald backward selection) regarding 28days-mortality.

Two regression analyses were restricted to the subgroup with TPTD monitoring. This allowed for analysing prediction by EVLWI in addtion to standard ARDS-scores.

In a first step, we included OI, Berlin, AECC, Murray_mod and EVLWI.

In a second step, we also included the general ICU-scores APACHE-II and SOFA in addition to OI and EVLWI.

Prevalence of TPTD-monitoring in about half of the patients allowed to analyse a potential impact of *„TPTD-monitoring per se“* with 28d-mortality as a major secondary endpoint. Necessarily, this analysis was performed *in the totality* of patients (49 patients with and 50 patients without TPTD-monitoring). For this analysis, we included APACHE-II, SOFA and TPTD-monitoring.

For comparison of baseline or other characteristics between groups, we used the Chi-square-test and the Wilcoxon-test for unpaired samples.

Due to the online documentation of all relevant data only few variables were missing due to technical or organizational reasons (e.g. absence from the ICU due to external examinations). In this case statistical tests were performed based on all measurements with valid data.

The sample size was calculated based on the assumption of a rate of correct prediction of 67% regarding 28-days mortality. This would require a study population of n=65 to demonstrate a significantly better prediction of the outcome compared to prediction “by chance” (67% vs. 50%) with p <0.05 and a statistical power of 80% (one group; dichotomous primary endpoint).

Assuming a drop-out rate (deaths, transfer within the first days) of 33% until day-4, n=100 patients were included.

All statistical anlyses were performed using IBM SPSS 26.

## Results

### Patients´ characteristics

Due to early transfer to another hospital and early discharge, for one patient final information on 28-d mortality was missing. Therefore, 99 complete data sets were finally analyzed.

Patients’ *baseline characteristics* on day 1 are shown in Table 2. 40.4% of the patients suffered from primary, 59.6% from secondary ARDS. Primary ARDS was defined as patients suffering from direct lung injury including pneumonia (bacterial, viral, fungal, or opportunistic), aspiration of gastric contents, pulmonary contusion, inhalation injury or patients with near drowning), wheras for patients with secondary ARDS patients no underlying causes for primary ARDS could be identified (e.g. sepsis of nonpulmonary source, nonthoracic trauma or hemorrhagic shock, pancreatitis, major burn injury, drug overdose, transfusion of blood products, cardiopulmonary bypass, reperfusion edema after lung transplantation or embolectomy) [9].

**Table 2:** Patients’ basic characteristics on day 1.

Parameter	All patients (n=99)	Primary ARDS (n=40)	Secondary ARDS (n=59)	p-value	Patients with TPTD (n=49)	Patients without TPTD (n=50)	p-value
Age (years)	62±14	63±14	62±14	0.697	60±14	65±13	0.080
Male sex (no; %)	54 (55%)	24 (60%)	30 (51%)	0.369	26 (53%)	28 (56%)	0.769
Height (1)	172±8	172±8.9	172±7	0.868	172±7	172±9	0.752
Weight (kg)	76±14	73±12	77±15	0.201	76±15	76±13	0.934
SOFA score	11±4	8.48±4.51	12.03±3.86	<0.001	11.45±4.58	9.76±4.24	0.070
APACHE-II score	22±7	21±7	22±7	0.528	22±8	21±7	0.456
P_a_O_2_/FiO_2_ (mmHg)	191±68	180±66	197±68	0.253	188±62	195±73	0.398
Oxygenation index (cmH_2_O/mmHg)	8.41±6.4	9.6±8.4	7.6±4.2	0.162	8.4±4.4	8.4±8.0	0.116

RDS: Acute Respiratory Distress Syndrome; APACHE-II: Acute Physiology And Chronic Health Evaluation; SOFA: Sequential Organ Failure Assessment; TPTD: transpulmonary thermodiltion

Patients with primary ARDS had a significantly lower SOFA score on day 1 (8.48 vs. 12.03; p<0.001).

Patients with PiCCO-monitoring showed a trend to higher SOFA-values compared to the patients without PiCCO (11.45 vs. 9.76; p=0.070).

### Mortality-analyses: All patients

28-days-mortality was 40 out of 99 (40.1%).

**Fig 1:**
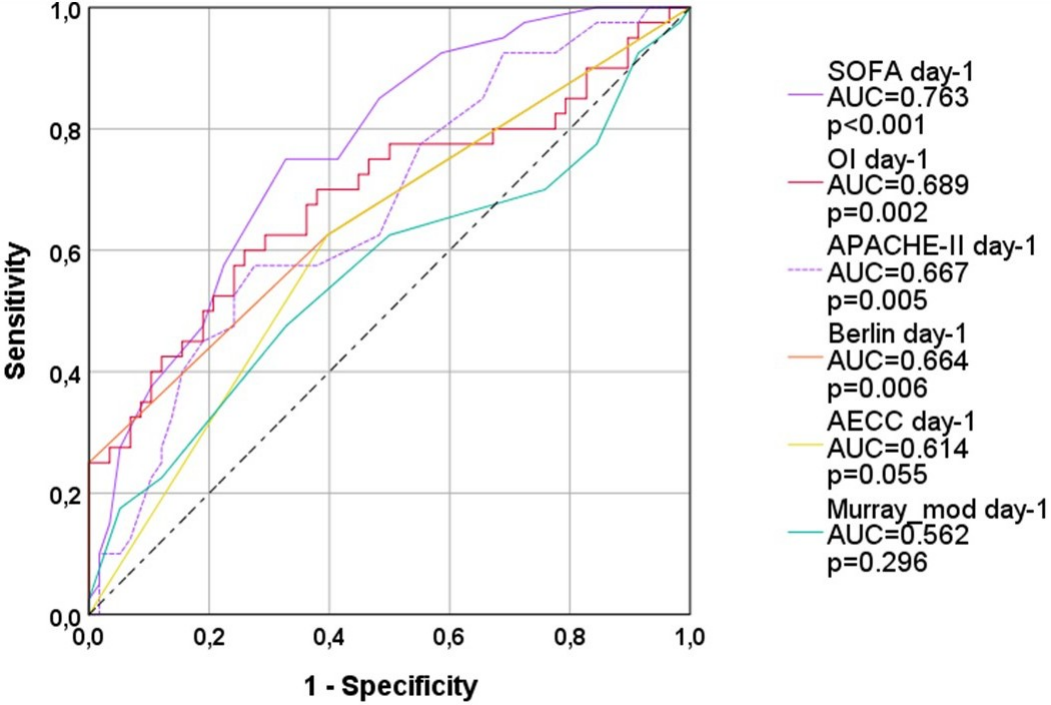
ROC-AUC regarding 28d-mortality (all patients; day 1). OI: oxygenation index; AECC: American European Consensus Conference; Murray_mod: modified Murray-score (sum of points without radiological points); AUC: area under the curve. APACHE-II: Acute Physiology And Chronic Health Evaluation; SOFA: Sequential Organ Failure Assessment.

On day-1, the largest ROC-AUC among the four respiratory scores was provided by OI (AUC=0.689; p=0.002; Fig 1; Table 3). Furthermore, the Berlin-definition was significantly associated with 28-days-mortality (AUC=0.664; p=0.006), whereas AECC and Murray_mod were not predictive.

**Table 3:** Prediction of 28-days-mortality by OI, Berlin-, AECC-definition and modified Murray-score: All patients.

Predictor	Day-1			Day-2			Day-3			Day-4			Mean-AUC
	AUC	95%-CI	p-value	AUC	95%-CI	p-value	AUC	95%-CI	p-value	AUC	95%-CI	p-value	
OI	0.689	0.576-0.801	0.002	0.632	0.513-0.752	0.034	0.603	0.470-0.736	0.134	0.577	0.439-0.716	0.280	0.625
Berlin	0.664	0.551-0.777	0.006	0.644	0.526-0.762	0.021	0.589	0.456-0.722	0.196	0.476	0.337-0.616	0.740	0.593
AECC	0.614	0.501-0.728	0.055	0.620	0.502-0.739	0.054	0.585	0.452-0.718	0.219	0.476	0.336-0.615	0.732	0.574
Murray_mod	0.562	0.441-0.683	0.296	0.582	0.459-0.704	0.192	0.620	0.489-0.751	0.082	0.514	0.375-0.654	0.841	0.577
Mean-AUC	0.632			0.620			0.599			0.511			

OI: Oxygenation Index; AECC: American European consenus conference; Murray_mod: modified Murray-score; AUC: Area under the curve; 95%-CI: 95% confidence interval

While the AUC for the APACHE-II-score (AUC=0.667; p=0.005; Table 4) was smaller than for OI, the SOFA-score had the largest AUC of all predictors (AUC=0.763; p<0.001) on day-1.

**Table 4:** Prediction of 28-days-mortality by SOFA-and APACHE-II-score: All patients and patients with PiCCO-monitoring on all four days (d1-d4).

Cohort/ Subgroup		Day-1			Day-2			Day-3			Day-4		
		AUC	95%-CI	p-value	AUC	95%-CI	p-value	AUC	95%-CI	p-value	AUC	95%-CI	p-value
All patients	SOFA	0.763	0.669-0.856	0.000	0.780	0.686-0.875	0.000	0.796	0.696-0.895	0.000	0.790	0.686-0.894	0.000
	APACHE-II	0.667	0.559-0.776	0.005	0.680	0.570-0.790	0.004	0.684	0.562-0.806	0.008	0.674	0.549-0.800	0.015
Patients with PiCCO	SOFA	0.774	0.637-0.912	0.002	0.775	0.638-0.912	0.002	0.755	0.610-0.901	0.005	0.712	0.545-0.878	0.033
	APACHE-II	0.627	0.459-0.795	0.155	0.614	0.451-0.776	0.203	0.599	0.432-0.766	0.275	0.649	0.473-0.826	0.132

AUC: Area under the curve; 95%-CI: 95% confidence interval; SOFA: Sequential Organ Failure Assessment; APACHE-II: Acute Physiology And Chronic Health Evaluation

**Fig 2:**
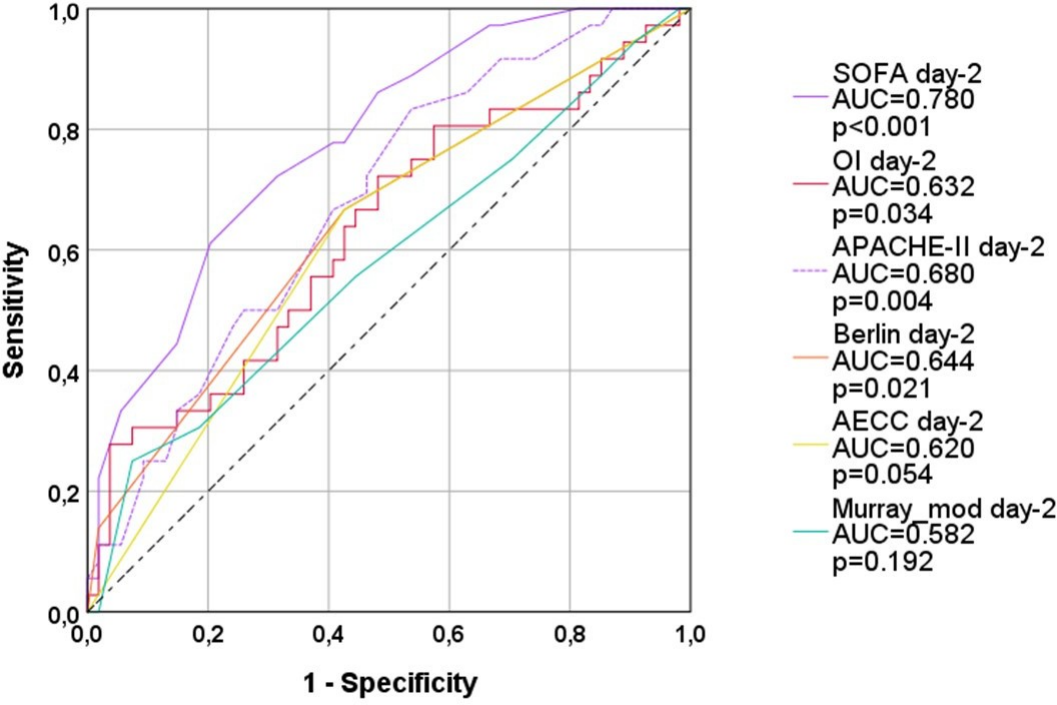
ROC-AUC regarding 28d-mortality (all patients; day 2). OI: oxygenation index; AECC: American European Consensus Conference; Murray_mod: modified Murray-score (sum of points without radiological points); AUC: area under the curve. APACHE-II: Acute Physiology And Chronic Health Evaluation; SOFA: Sequential Organ Failure Assessment.

On day-2, OI (AUC=0.632; p=0.034) and Berlin-definition (AUC=0.644; p=0.021; Fig 2; Table 3) predicted 28-days-mortality with significant p-values, but poor ROC-AUCs. AECC-definition and Murray_mod were not predictive.

SOFA (AUC=0.780; p<0.001; Table 4) predicted 28-days-mortality better than the APACHE-II (AUC=0.680; p=0.004).

**Fig 3:**
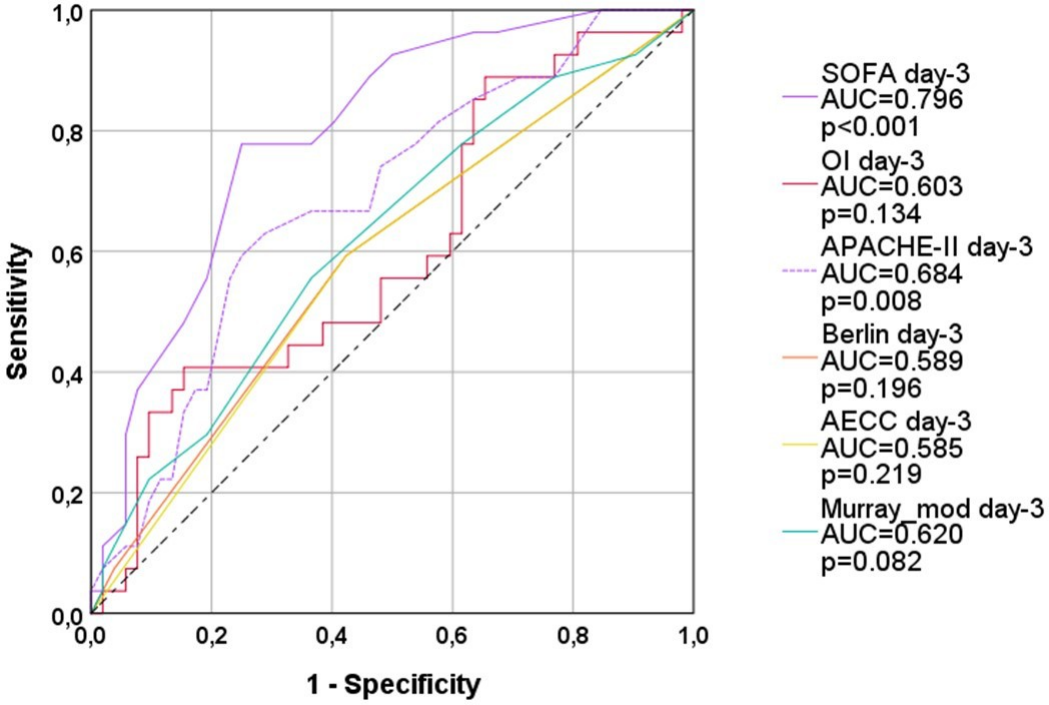
ROC-AUC regarding 28d-mortality (all patients; day 3). OI: oxygenation index; AECC: American European Consensus Conference; Murray_mod: modified Murray-score (sum of points without radiological points); AUC: area under the curve. APACHE-II: Acute Physiology And Chronic Health Evaluation; SOFA: Sequential Organ Failure Assessment.

**Fig 4:**
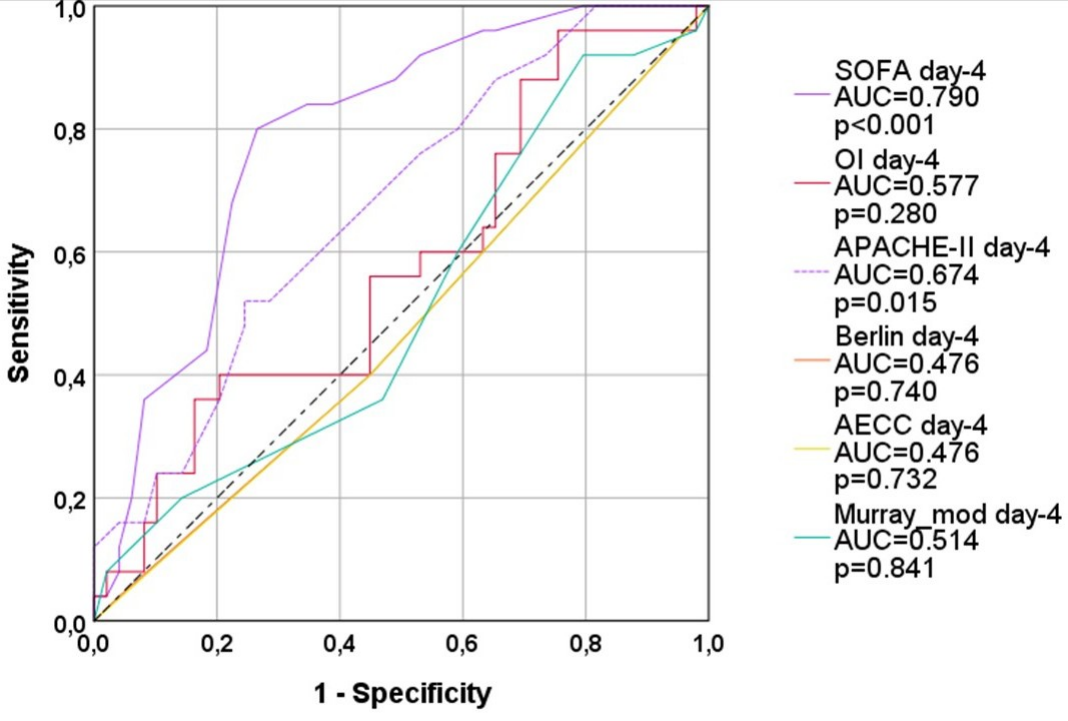
ROC-AUC regarding 28d-mortality (all patients; day 4). OI: oxygenation index; AECC: American European Consensus Conference; Murray_mod: modified Murray-score (sum of points without radiological points); AUC: area under the curve. APACHE-II: Acute Physiology And Chronic Health Evaluation; SOFA: Sequential Organ Failure Assessment.

On day-3 and on day-4 none of the four ARDS-scores significantly predicted 28-days-mortality (Figs 3 and 4; Table 3).

SOFA (AUC=0.796; p<0.001) provided larger AUCs than APACHE-II (AUC=0.684; p=0.008) on day-3 and day-4 (AUC=0.790; p<0.001 vs. AUC=0.674; p=0.015; Table 4).

OI was the best predictor among the respiratory scores with a mean AUC of 0.625 (see Table 3), whereas the mean AUCs for all other scores were below the critical threshold of 0.6.

Regarding the *timing of prognosis*, the best prediction of 28-days-mortality was found on day-1 (mean ROC-AUC=0.632) and day-2 (mean ROC-AUC=0.620; see Table 3), whereas mean ROC-AUCs were below 0.6 on day-3 and on day-4.

### Mortality-analyses: Subgroup with TPTD-monitoring (n=49)

**Fig 5:**
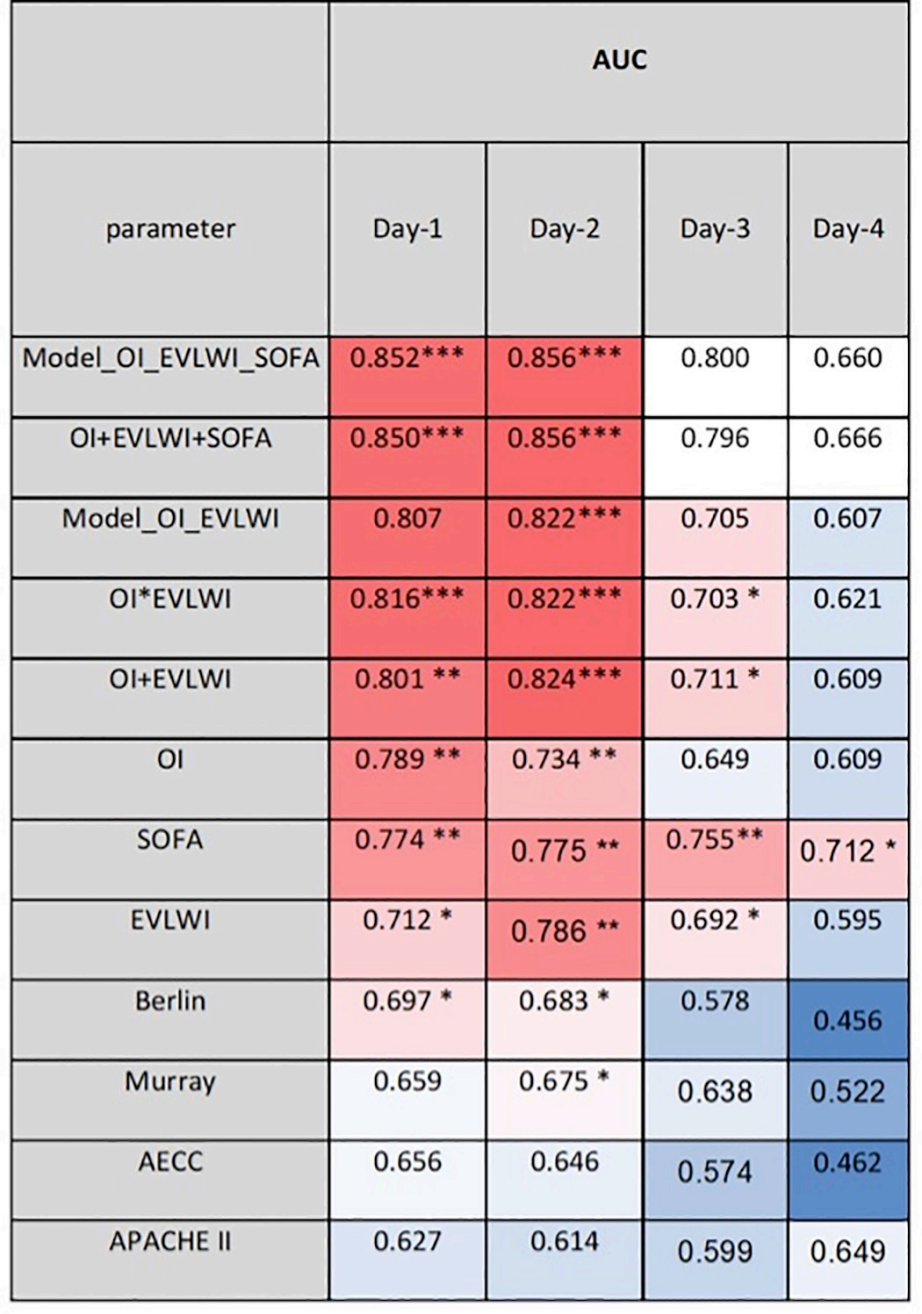
Thermoplot illustrating ROC-AUCs of single parameters, scores and combined models within four days after intubation in patients with PiCCO-monitoring. Y-axis ranges from 0.5 (worthless for prediction; intense blue) to 1.0 and 0.0 (best prediction; intense red). AUC: area under the curve.OI: oxygenation index; EVLWI: Extra-vascular Lung Water Index; AECC: American European Consensus Conference; Murray_mod: modified Murray-score (sum of points without radiological points); AUC: area under the curve. APACHE-II: Acute Physiology And Chronic Health Evaluation; SOFA: Sequential Organ Failure Assessment.

Among patients with TPTD-monitoring, EVLWI had the best predictive capacities (Fig 5; Table 5): EVLWI provided the largest ROC-AUC on day-2 (AUC=0.786; p=0.001). EVLWI was also predictive on day-1 (AUC=0.712; p=0.018). EVLWI was the only parameter predicting 28-days-mortality on day-3 (AUC=0.692; p=0.035). The mean AUC for day-1 to day-4 was 0.696 for EVLWI (Table 5). OI was predictive on day-1 (AUC=0.789; p=0.001) and on day-2 (AUC=0.734; p=0.009), but not on day-3 and day-4. Next to EVLWI, OI provided the largest mean ROC-AUC (0.695; Table 5).

**Table 5:** Prediction of 28-days-mortality by OI, Berlin-, AECC-definition, Murray-score and EVLWI: Patients with PiCCO-monitoring on all four days (d1-d4).

	Day-1			Day-2			Day-3			Day-4			Mean-AUC
	AUC	95%-CI	p-value	AUC	95%-CI	p-value	AUC	95%-CI	p-value	AUC	95%-CI	p-value	
OI	0.789	0.647-0.931	0.001	0.734	0.588-0.880	0.009	0.649	0.479-0.818	0.102	0.609	0.425-0.794	0.270	0.695
Berlin	0.697	0.535-0.860	0.027	0.683	0.518-0.847	0.041	0.578	0.401-0.754	0.393	0.456	0.265-0.646	0.655	0.604
AECC	0.656	0.494-0.819	0.080	0.646	0.481-0.811	0.101	0.574	0.398-0.751	0.413	0.462	0.269-0.654	0.699	0.585
Murray_mod	0.659	0.499-0.819	0.075	0.675	0.513-0.838	0.049	0.638	0.468-0.808	0.129	0.522	0.335-0.709	0.823	0.624
EVLWI	0.712	0.562-0.862	0.018	0.786	0.653-0.919	0.001	0.692	0.531-0.852	0.035	0.595	0.412-0.778	0.340	0.696
Mean-AUC	0.703			0.705			0.626			0.529			

OI: Oxygenation-index; AECC: American European consenus conference; Murray_mod: modified Murray-score; AUC: Area under the curve; 95%-CI: 95% confidence interval

The Berlin-definition was associated with 28-days-mortality on day-1 (AUC=0.697; p=0.027) and on day-2 (AUC=0.683; p=0.041), but not on day-3 and day-4. The mean ROC-AUC over four days (AUC=0.604) was substantially smaller for the Berlin-definition than for EVLWI and OI.

Murray_mod was predictive only on day-2 (AUC=0.675; p=0.049). The mean ROC-AUC (AUC=0.624) for Murray_mod was slightly larger than for the Berlin-definition.

The AECC-definition did not predict 28-days-mortality on any day and provided the smallest mean ROC-AUC (AUC=0.585).

Regarding the *timing of prognosis*, as for the totality of patients, the best prediction of 28-days-mortality was found on day-1 (mean AUC=0.703) and day-2 (mean AUC=0.705; Table 5; Fig 5).

### Multivariate analysis including ARDS-scores and EVLWI

In binary regression analysis regarding 28-days-mortality including OI, Berlin, AECC, Murray_mod and EVLWI, only EVLWI (p=0.007) and OI (p=0.006) were independently associated with 28-days-mortality.

The B- (-0.105 and -0.124), Wald- (7.294 and 7.435) and p-values (p=0.007 and p=0.006) in the regression equation were similar for EVLWI and OI. This implicates that both parameters had a similar impact and similar absolute values in the model. These findings suggests that simple addition (OI+EVLWI) or multiplication (OI*EVLWI) could provide similar predictive capacities as the more complex model (prediction formula: 2.917-0.124*OI-0.105*EVLWI).

As shown in Fig 5 the complete regression formula as well as the simplified formulas EVLWI+OI and EVLWI*OI outscored all ARDS-scores and single parameters on all four days.

The best day to predict 28-days-mortality by the combination of EVLWI and OI was day-2 with ROC-AUCs of up to 0.824.

A cut-off of 19 for the sum of EVLWI (mL/kg)772 + OI (cmH_2_O/mmHg) on day-2 provided a sensitivity of 71% and a specificity of 79% to predict 28-days-mortality.

As for the totality of patients, SOFA better predicted mortality compared to APACHE-II. The largest AUCs for SOFA and APACHE-II were found on day-2 (SOFA: AUC=0.775; APACHE-II: AUC=0.614). However, the AUCs on day-2 were smaller than for the combinations of EVLWI and OI (0.822-0.824; Fig 5; Table 5).

**Fig 6:**
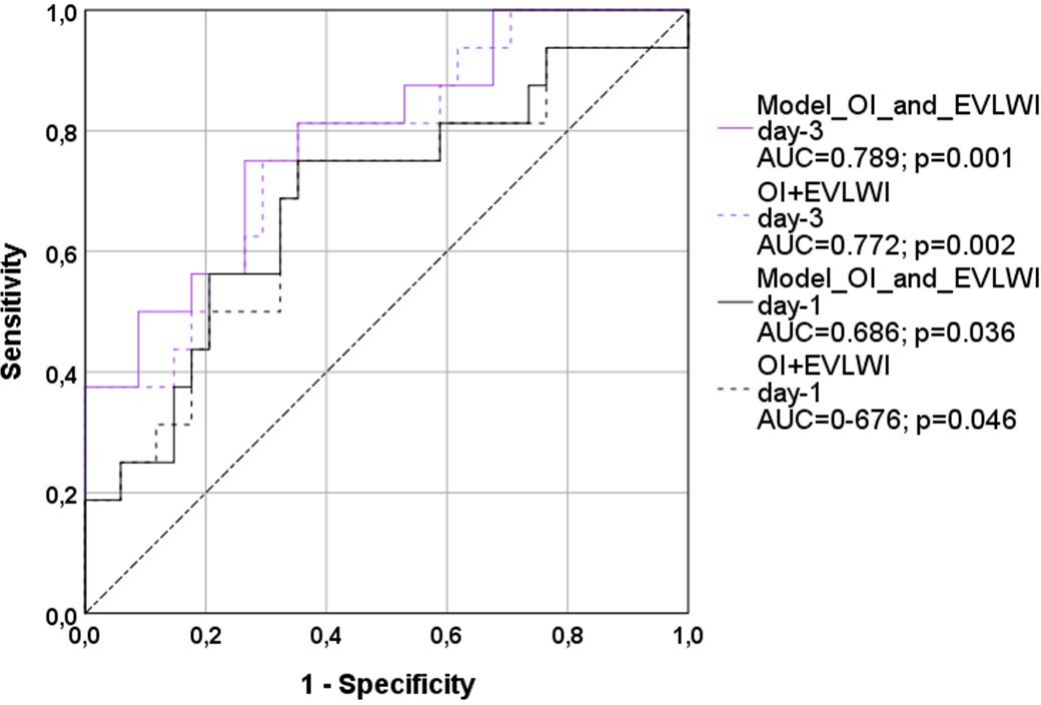
Validation-study (dataset (n=50) from reference [20]): ROC-AUC regarding mortality. The models OI_and_EVLWI were calculated from the regression formula derived from this study (evaluation study). OI: oxygenation index; EVLWI: Extra-vascular Lung Water Index. AUC: area under the curve.

For *validation*, we re-analyzed an independent dataset from a previous study [20]. This analysis demonstrated significant and comparable ROC-AUCs for „OI+EVLWI“ (day-1: AUC=0.676; p=0.046; day-3: AUC=0.772; p=0.002; Fig 6) and the more complex „OI and EVLWI-model“ (day-1: AUC=0.686; p=0.036; day-3: AUC=0.789; p=0.001).

### Prediction of mortality: Combination of SOFA with EVLWI and OI

In the next step, we performed binary regression analysis regarding 28-days-mortality including SOFA-, APACHE-II-score, OI and EVLWI. While APACHE-II did not independently predict 28d-mortality, EVLWI (p=0.018), OI (p=0.022) and SOFA (p=0.015) were independent predictors of 28-days-mortality.

Finally, *univariate comparison of patients with and without PiCCO-monitoring* showed a trend to lower mortality in patients with PiCCO-monitoring (16/49 (33%) vs. 24/50 (48%); p=0.12) despite a trend to a higher baseline-SOFA-score (11.45±4.58 vs. 9.76±4.24; p=0.070).

In binary regression analysis including “use of PiCCO-monitoring”, SOFA and APACHE-II, only “use of PiCCO-monitoring” (p=0.007) and lower SOFA-score (p<0.001) were independently associated with a lower 28-days-mortality.

## Discussion

Protracted or even non-recognititon of ARDS contributes to its high mortalitiy. This might be due to low nurse-to-patient ratios, low physician-to-patient ratios, older patient age, higher p_a_O_2_/F_i_O_2_ ratio, and the absence of of pneumonia or pancreatitis. In a recent trial, all these factors were independently associated with higher probability of non-recognition of ARDS [1]. However, early recognition and grading of ARDS is crucial, since the effectiveness of several therapeutic measures depends on their early initiation [2, 4, 6, 10, 36]. This also applies to ECMO [28, 37].

Our analyses regarding timing and predictors of 28-days-mortality showed the following results:

1. OI better predicts 28-days-mortality compared to Berlin-definition, AECC and Murray_mod.
2. The best predictive capacities were found within the first two days after intubation.
3. EVLWI is a strong and independent predictor of 28-days-mortality.
4. The combination of EVLWI and OI further increases the predictive capacities of each parameter alone. „OI+EVLWI“ provides larger ROC-AUCs than SOFA and APACHE-II on the first two days.
5. SOFA better predicted 28-days-mortality than APACHE-II.
6. EVLWI, OI and SOFA were independently associated with 28-days-mortality.
7. Furthermore, TPTD-monitoring was independently associated with a lower 28-days-mortality.

Similar to previous studies, we found poor prognostic capacities of predictors mainly based on p_a_O_2_/F_i_O_2_. The predictive capacities of the Berlin-definition were poor even in the primary validation-study: the ROC-AUC was only slightly better compared to the AECC-definition (AUC 0.577 vs. 0.536) and below the minimum threshold of 0.6 [33]. Limited predictive capacities of AECC, Berlin and p_a_O_2_/F_i_O_2_ were found in numerous more recent trials [32, 38–41].

Also the Murray/LIS-score is strongly driven by p_a_O_2_/F_i_O_2_. It includes four categories of p_a_O_2_/F_i_O_2_, PEEP, compliance and radiological findings. Among these four parameters, only p_a_O_2_/F_i_O_2_ and PEEP were significantly different between survivors and non-survivors in a recent study [34].

Several reasons for the poor performance of p_a_O_2_/F_i_O_2_, AECC, Berlin and Murray need to be discussed. The cut-offs of 100, 175, 200, 225 and 300mmHg used in these scores are arbitrary and poorly validated. Furthermore, p_a_O_2_/F_i_O_2_ strongly dependends on ventilatory data including PEEP, inspiration/exspiration-ratio, driving-pressure and even F_i_O_2_ itself. Inclusion of minimum (Berlin) or categorized (Murray; [17] information of PEEP did not substantially improve prediction compared to AECC. Finally, all three scores do not account for the non-linear relationship of p_a_O_2_ and F_i_O_2_: As shown by Allardet-Servent and co-workers [42], p_a_O_2_/F_i_O_2_ strongly increases with higher values of F_i_O_2_ [42].

The strong performance of OI in our study is in line with several previous studies [19, 30, 32, 38, 43–46]. Incorporation of P_maw includes substantial additional information, since P_maw in addition to p_a_O_2_/F_i_O_2_ reflects PEEP, inspiration/expiration-ratio, peak-pressure, delta-pressure and ventilation-mode (assisted vs. controlled). The strong improvement of prediction by inclusion of P_maw is further emphasized by the strong performance of the oxygenation *saturation* index [47] in several recent studies [38, 48–50]. OSI replaces p_a_O_2_ by percutaneous oxygen saturation:

$$OSI\;=\;100*P\_maw*F_{i}O_{2}/S_{a}O_{2}$$

Best prediction of outcome on *day-2* in our study is in line with some [30, 31, 41], but not all of the few studies performing sequential prediction of mortality in ARDS. The study by Balzer et al. [32] analyzed prediction of mortality on day-1 to day-7 in 442 patients. It showed increasing predictive capacities from day-1 to day-3 and comparable ROC-AUCs from day-3 to day-7. However, two thirds of the patients extracted from a seven-year-database had been transferred from other hospitals, and 58% were treated with extracorporeal lung-assist after transfer. Both, transfer with previous ventilation and extracorporeal lung-support might have influenced the best time of prediction.

The strong performance of EVLWI in our study is supported by previous data, since EVLWI has been associated with mortality in numerous studies [20, 25, 51–56].

Some of these studies also demonstrated *independent* association of EVLWI with mortality in addition to APACHE-II [20], SOFA [55, 57] and SAPS [56]. Interestingly, in the studies by Mallat [57] and Craig [55], EVLWI and SOFA had similar odds ratios in the multivariate analyses. These data suggest a similar impact in a combined model which supports our finding that EVLWI, OI and SOFA were independently and to similar degree associated with 28-days-mortality.

While the combination of OI and EVLWI might be usefull for *selection* of patients for ECMO, SOFA might be used as an *exclusion criterion* for ECMO: Several ECMO registries and EOLIA suggest that even early ECMO does not improve outcome in patients with high SOFA-scores [3, 8, 58–61].

Finally, the finding that the early use of TPTD-monitoring „per se“ independently reduced mortality in patients with ARDS is of high interest.

As expected according to the local standard, patients with PiCCO-monitoring available within 24h after intubation showed a trend to more severe organ impairment (mean SOFA 11.45 vs. 9.76; p=0.070).

A recent study suggests increases in mortality of about 7% for each SOFA-point [62]. Accordingly, mortality should be about 13% higher in our patients with PiCCO-monitoring. However, it was 15% lower (33% vs. 48%). This reduction of the predicted mortality-difference by 28% by advanced monitoring “per se” should be interpreted with caution, although these findings are in line with previous studies suggesting potentially beneficial effects of PiCCO-monitoring with [63–67] and without [68, 69] pre-defined algorithms. Similar to our study, a RCT in patients with ARDS and septic shock demonstrated a comparable mortality between groups despite a 17% percent higher predicted mortality according to SOFA and APACHE-II in the PiCCO-group compared to the controls [65, 66].

### Strengths and practical applications

This is one of few studies comparing daily prediction of mortality in ARDS by AECC, Berlin, Murray/LIS and OI over four days after intubation. Availability of TPTD-monitoring in about 50% of the patients allowed for comparing these predictors to EVLWI in a substantial subgroup, and for analyzing the impact of PiCCO-monitoring per se.

The usefulness of EVLWI and OI could be validated in an independent validation group.

### Limitations

Evaluation and validation were performed in a single center. TPTD-data were obtained in only half of the patients. Furthermore, prediction of a high mortality with high sensitvity and specifity by a single or few parameters in a mono-centric cohort rarely justifies limitation of therapy in an individual patient. However, in addition to a practical use (better allocation to different treatment options; in particular allocation of patients „at need“ to limited ressources) predictors help to compare patient populations in studies or and audits.

Another limitation is our „pragmatic“ approach with crossover–comparison of several predictors of 28-days on four different days. This might induce a kind of „immortality bias“: Since a substantial number of patients died or was transferred within the first three days, the basis of observation and the number of patients analysed on day-4 were different from day-1. From a statistician´s viewpoint, one could overcome this problem by a limitation of the anaylsis to patients surviving at least to day-5. However, this would eliminate half of the non-survivors (20 out 40) who died within the first four days. Regarding better allocation of patients to early treatment options such as ECMO, this approach would eliminate the most interesting subgroup of our study. On the other hand, this approach would focus on predictors of *late* mortality. Ex-post analyses of this study demonstrate that the SOFA-score best predicted late mortality, whereas P/F-ratio, AECC- and Berlin-definition and modified Murray-score were poor predictors (data not shown). Next to SOFA-score, the largest AUCs to predict death after day-4 were provided by Oxygenation-Index (AUC=0.700; p=0.008) on day-1, and by EVLWI on day-2 (AUC0.751; p=0.010) in the subgroup of patients with PiCCO-monitoring.

## Conclusions

Prognosis of ARDS-patients can be established within the first two days after intubation.

EVLWI, OI and SOFA were the best predictors. Similar cut-offs and numerical values facilitate their use in simple models resulting from addition of the raw values.

TPTD-monitoring „per se“ was independently associated with reduced mortality.

## Abbreviations

AECC: American European Consensus Conference
AOI: Age-adjusted oxygenation index
APACHE: Acute physiology and chronic health evaluation
ARDS: Acute respiratory distress syndrome
AUC: Area under the curve
ECMO: Extra-corporeal membrane oxygenation
EVLWI: Extravascular lung water index
F_i_O_2_: Fraction of inspired oxygen
I/E: Inspiration/exspiration
ICU: Intensive care unit
LIS: Lung injury score
MV: Mechanical ventilation
NMBA: Neuro-muscular blocking agent
OI: Oxygenation index
OR: Odds ratio
OSI: Oxygenation saturation index
P_maw: Mean airway pressure
P_plat: Plateau pressure
p_a_O_2_: Arterial partial oxygen pressure
PEEP: Positive end-expiratory pressure
PP: Prone positioning
RCT: Randomized controled trial
ROC: Receiver operating caracteristic
SaO2: Arterial oxygen saturation
SAPS: Simplified acute physiology score
SOFA: Sequential organ failure assesment
TPTD: Transpulmonary thermodilution
